# Effect of deviated nasal septum on Eustachian tube dysfunction: a systematic review and meta-analysis

**DOI:** 10.1017/S0022215125000325

**Published:** 2025-07

**Authors:** Muhammad Ozair Awan, Dimitrios Spinos, Muzamil Hussain, Jameel Muzaffar, Aman Khanna, Asad Qayyum, Remo Accorona, Haissan Iftikhar

**Affiliations:** 1 Medical College, Aga Khan University Hospital, Karachi, Pakistan; 2 Department of Otolaryngology Head and Neck Surgery, University Hospital Birmingham, Birmingham, UK; 3 Department of Otolaryngology, University Hospital Birmingham, Birmingham, UK; 4 University Hospitals Northamptonshire, UK; 5 Unit of Otorhinolaryngology, ASST Grande Ospedale Metropolitano Niguarda, Milano, Italy; 6 Department of Otolaryngology, University Hospitals Northamptonshire, Northamptonshire, UK

**Keywords:** deviated nasal septum, Eustachian tube dysfunction, nasal surgery, septoplasty

## Abstract

**Objective:**

A range of chronic ear complaints may be attributed to Eustachian tube dysfunction. Eustachian tube dysfunction secondary to a deviated nasal septum has been described in several clinical studies, with symptomatic improvement demonstrated following septoplasty. However, uncertainty exists as to the size of the effect and consistency between studies.

**Methods:**

Electronic searches were carried out of Pubmed, Embase and the Cochrane Library for adult patients with complaints of nasal obstruction and/or impairment and/or complaints of ear fullness undergoing nasal surgery.

**Results:**

Seven studies met the inclusion criteria. Studies evaluated the effect of nasal surgery on Eustachian tube dysfunction using a variety of outcomes, including Eustachian tube function tests, the Eustachian Tube Dysfunction Questionnaire-7, tympanometry and Nasal Obstruction Symptom Evaluation scores. The results demonstrated the positive impact of nasal surgery on various outcomes related to Eustachian tube dysfunction.

**Conclusion:**

Nasal surgery has been demonstrated to have promising results as a therapeutic option for patients with Eustachian tube dysfunction and a deviated nasal septum, offering significant symptom relief and improved quality of life. Through the integration of the treatment of nasal symptoms in the management of Eustachian tube dysfunction, clinicians can adopt a comprehensive approach to addressing the underlying pathologies contributing to Eustachian tube dysfunction.

## Introduction

A variety of chronic middle-ear symptoms and their respective pathologies have been attributed to irregularities in middle-ear cleft ventilation, with Eustachian tube dysfunction considered to be a leading cause of pathologies, including otitis media with effusion in children and chronic otitis media in adults.^1^^–^^4^ Eustachian tube dysfunction can be broadly classified into obstructive, barochallenge or patulous dysfunction, and commonly results in symptoms such as a popping sensation, hearing loss, tinnitus and otalgia.^3^^,^^5^

There is no ‘gold standard’ test for Eustachian tube dysfunction, but a variety of tests are used in clinical and research settings, for example tubotympanic aerodynamic graphs, tubotympanometry, sonotubometry and Toynbee manoeuvres.^5^ The most used clinical test for the diagnosis of Eustachian tube dysfunction is impedance audiometry, commonly termed tympanometry, in which a tympanogram is produced reporting the tympanic membrane compliance, a measure that is predominantly a function of middle-ear pressure and is used as a proxy for Eustachian tube dysfunction. In addition to this, patient-reported outcome measures such as the validated Eustachian Tube Dysfunction Questionnaire-7 can help to diagnose Eustachian tube dysfunction and guide the selection of treatment options.^6^

Multiple theories have been described to explain Eustachian tube dysfunction. One such theory is compression of the opening of the Eustachian tube in the nasopharynx either by a large adenoid pad or by a nasopharyngeal neoplasm, leading to serous otitis media and hearing loss.^1^^,^^7^ Recently, there has been an emergence of evidence supporting chronic rhinosinusitis as an underlying cause of Eustachian tube dysfunction. This can be explained by the concept of the unified airway, with nasal inflammation affecting the Eustachian tube in the nasopharynx as well the nose and paranasal sinuses.^7^

Other authors have suggested that changes in nasal airflow have the potential to cause Eustachian tube dysfunction.^1^^–^^4^^,^^8^ Several potential mechanisms have been suggested, such as an increase in tubal mucosal viscosity and surface tension caused by altered air currents, in a similar manner to what happens in the case of nasal crusting secondary to a septal perforation, or deposition of irritants within the region of tubal opening due to the aberrant airflow, leading to stimulation of the local autonomic innervation.^1^ This disturbance to airflow can be attributed to a deviated nasal septum, turbinate hypertrophy or a septal perforation, with a deviated nasal septum being the most common cause.^1^^,^^7^ Several procedures to correct the deviated nasal septum have been described, septoplasty being most common one.^9^

Although Eustachian tube dysfunction as the result of a deviated nasal septum has been described in several studies, so far there has been no systematic review to explore the potential effect size across these studies. This review aimed to report changes in Eustachian tube function or middle-ear ventilation in patients with a deviated nasal septum undergoing septoplasty.

## Methods

### Search strategy

The Preferred Reporting Items for Systematic Reviews and Meta-Analyses Guidelines^10^ were used to conduct this systematic review following registration in the International Prospective Register of Systematic Reviews (CRD 42023412013). Records were identified via the databases Pubmed, Embase and the Cochrane Library, with searches conducted on 8 March 2024 using the following search terms: (retraction pocket or tympanic retraction or Eustachian tube dysfunction or Eustachian dysfunction or chronic otitis media or otitis media with effusion or middle-ear ventilation or middle-ear ventilation dysfunction or aural fullness) and (septoplasty or functional endoscopic sinus surgery or functional endoscopic sinus surgery (FESS) or turbinoplasty or nasal surgery or rhinoplasty or septorhinoplasty or nose surgery or sinus surgery). Any conflicts that arose during this process were resolved via discussion with another author (DS). The search was expanded from the beginning of time to May 2024; language was restricted to English.

### Data extraction and management

Data were extracted from the included studies by two authors (MOA and MHH). Extracted data parameters included author names, year, country and journal of publication, study design, population number and age, inclusion and exclusion criteria, intervention delivered, follow-up duration and outcomes measured. In addition, references from the selected full-text articles were screened for potential papers that could be included. In case of any disagreement between the two authors, the opinions of a third author (HI) were sought to resolve the concerns. Data extraction was conducted following final study selection on a pre-formulated data extraction sheet recording study parameters as well as outcome measures.

### Population, Invervention, Comparison and Outcome

The study population comprised adult patients with complaints of nasal obstruction and/or impairment and/or complaints of ear fullness. The intervention was nasal surgery to correct nasal airflow, including septoplasty, submucosal diathermy and turbinate surgery. A comparison was made with no surgical intervention. Outcomes were recorded using the Eustachian tube function tests, the Eustachian Tube Dysfunction Questionnaire-7, tympanometry and Nasal Obstruction Symptom Evaluation scores.

The exclusion criteria included studies assessing patients with congenital malformations of the ear or nose, otosclerosis, ossicular chain dysfunction, history of previous nasal or ear surgical procedures, current or recent nasal or otologic infections, malignant lesions and/or previous radiotherapy of the head and neck region.

### Statistical and risk of bias analysis

**Figure 1.**
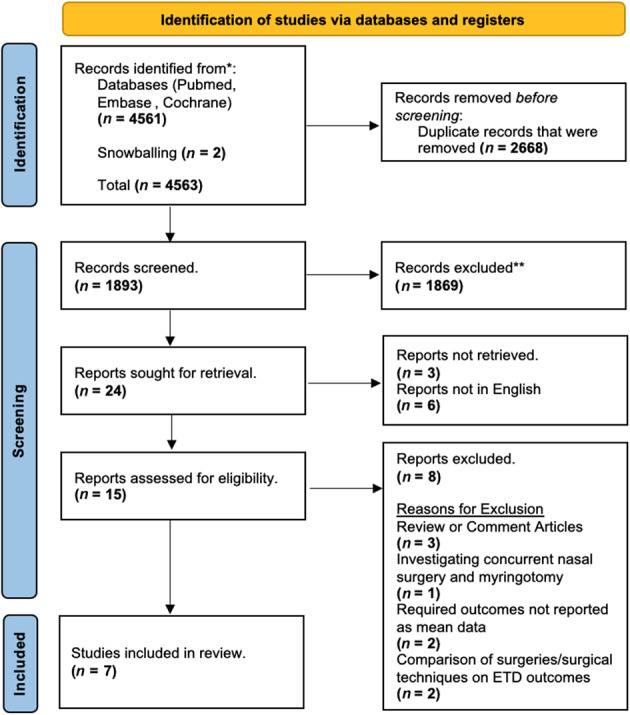
PRISMA DNS and ETD.

Quality assessment was carried out using the National Institute of Health (NIH) Quality Assessment Tool for before–after (pre–post) studies with no control group (Table 1). Risk of bias assessment was performed with the Cochrane RoB2 tool^11^ (Figure 1). Meta-analysis was conducted using Review Manager (RevMan 5.4.1; Cochrane, UK). A random effects model was used throughout, and outcomes were reported as mean differences and odds ratios where appropriate.

**Table 1.** Natioanl Institute of Health quality assessment for before and after (pre–post) studies with no control group

First author (year)	Response												Total score (out of 11 (%))	Quality rating
	Q1	Q2	Q3	Q4	Q5	Q6	Q7	Q8	Q9	Q10	Q11	Q12		
Alshareef (2022)^8^	Y	Y	Y	Y	CD	Y	Y	N	Y	N	N	NA	7 (63.6)	Moderate risk of bias
Salvinelli (2005)^4^	Y	Y	Y	Y	CD	Y	Y	N	Y	N	N	NA	7 (63.6)	Moderate risk of bias
Lima (2022)^3^	Y	Y	Y	Y	CD	Y	Y	N	Y	Y	N	NA	8 (72.7)	Moderate risk of bias
Lee (2022)^2^	Y	Y	Y	Y	CD	Y	Y	N	N	Y	N	NA	7 (63.6)	Moderate risk of bias
Kaya (2018)^12^	Y	Y	Y	Y	CD	Y	Y	N	Y	Y	N	NA	8 (72.7)	Moderate risk of bias
Akyildiz (2017)^13^	Y	Y	Y	Y	CD	Y	Y	N	Y	N	N	NA	7 (63.6)	Moderate risk of bias
Daum (2024)^14^	Y	Y	Y	Y	CD	Y	Y	N	Y	Y	N	NA	8 (72.7)	Moderate risk of bias

Q = question; Y = yes; CD = Cannot be determined; N = no; NA = Not applicable

## Results

### Search results and study characteristics

**Figure 2.**
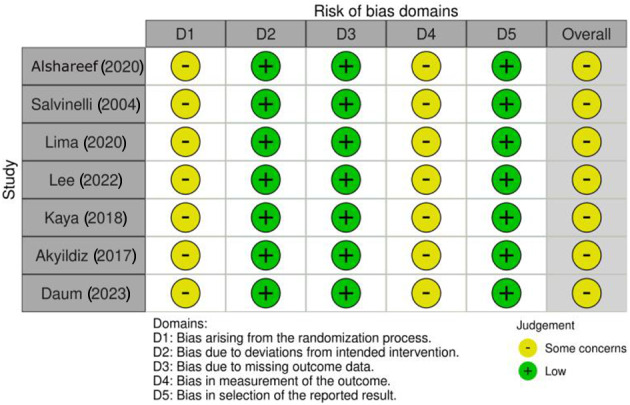
Risk of Bias Assessment.

Our initial search resulted in a yield of 1961 results. Reference lists of included studies were searched and a further two papers were identified via snowball methodology, bringing the total yield to 1963 articles. Initial screening was conducted independently by two authors (MOA and MHH), followed by full-text review, yielding 15 papers. A total of seven articles^2^^–^^4^^,^^8^^,^^12^^–^^14^ reported quantitative findings, making them eligible for meta-analysis (Table 2 and Figure 2). Each study evaluated the effect of nasal surgery on Eustachian tube dysfunction using various measures of outcomes, such as Eustachian tube function tests, the Eustachian Tube Dysfunction Questionnaire-7, tympanometry and Nasal Obstruction Symptom Evaluation scores (Table 3).

**Table 2.** Characteristics of included studies

First author (year)	Journal	Country of publication	Study design	Intervention	Participants	Population age (years)
Alshareef (2022)^8^	*Zagazig University Medical Journal*	Egypt	Clinical trial	Septoplasty + inferior turbinectomy	18	Mean: 33.1 ± 8.8
Salvinelli (2005)^4^	*Clinical Otolaryngology*	Italy	Prospective cohort	Septoplasty and/or inferior turbinectomy	40	Mean: 39
Lima (2022)^3^	*Acta Otorrinolaringologica Espanola*	Portugal	Prospective study	Septoplasty	35	Median (IQR): 40 (19)
Lee (2022)^2^	*Rhinology*	Republic of Korea	Retrospective cohort	Group A = septoplasty alone; group B = FESS alone; group C = septoplasty + FESS	Group A = 45 Group B = 106 Group C = 42 Total = 193	Mean: 48.19 ± 18.56
Kaya (2018)^12^	*Turkish Archives of Otorhinolaryngology*	Turkey	Prospective RCT	Septoplasty	50	Mean: 32.9 ± 9
Akyildiz (2017)^13^	*The Journal of Craniofacial Surgery*	Turkey	Prospective study	Septoplasty	Septoplasty group = 25 Control group = 25	Median (min–max): septoplasty group 33 (15−76); control group 27 (24−67)
Daum (2024)^14^	*Allergy and Rhinology*	USA	Retrospective chart review	Septoplasty or inferior turbinectomy	97	Mean: 39.6 ± 16

IQR = interquartile range; FESS = functional endoscopic sinus surgery; RCT = randomised controlled trial.

**Table 3.** Outcomes assessed in included studies

First author (year)	ETF tests	ETDQ−7	Tympanogram	NOSE score
Alshareef (2022)^8^	Yes	No	Yes	No
Salvinelli (2005)^4^	Yes	No	No	No
Lima (2022)^3^	Yes	Yes	No	Yes
Lee (2022)^2^	No	Yes	No	No
Kaya (2018)^12^	No	No	No	Yes
Akyildiz (2017)^13^	Yes	No	No	No
Daum (2024)^14^	No	Yes	No	No

ETF = Eustachian tube function; ETDQ-7 = Eustachian Tube Dysfunction Questionnaire-7; NOSE = Nasal Obstruction Symptom Evaluation.

### Eustachian tube function tests

Four studies utilised Eustachian tube function tests to assess the efficacy of surgical interventions (Table 4). Alshareef *et al*.^8^ utilised the Valsalva and Toynbee manoeuvres and reported pre-operative ‘good’ outcomes in 8 participants (44.4 per cent), which increased to 14 (77.8 per cent) after 1 month and further to 17 (94.4 per cent) after 6 months follow up post-surgery. However, Alshareef *et al*. did not report the exact Eustachian tube function test values used to determine a ‘good’ or ‘poor’ outcome in the test. Similarly, Salvinelli^4^ also utilised the Valsalva and Toynbee manoeuvres, and reported that pre-operative ‘good’ outcomes increased from 9 participants (22.5 per cent) to 17 participants (42.5 per cent) 15 days post-intervention, to 19 participants (47.5 per cent) 30 days post-intervention and to 32 participants (80 per cent) 90 days post-intervention. They reported that a tympanometric peak pressure shift of less than 10 daPa between pre- and post-intervention groups was recorded as a ‘poor’ outcome, while a shift of 10 daPa or more was recorded as a ‘good’ Eustachian tube function outcome.

**Table 4.** Eustachian tube function outcomes

First author (year)	Measurement tool	Time point	Poor outcome (*n*)	Good outcome (*n*)
Alshareef (2022)^8^	Valsalva and Toynbee manoeuvres	Pre-operative	20	16
		1 month post-operative	8	28
		6 months post-operative	2	34
Salvinelli (2005)^4^	Valsalva and Toynbee manoeuvres	Pre-operative	31	9
		15 days post-operative	23	17
		30 days post-operative	21	19
		90 days post-operative	8	32
Kaya (2018)^12^	Valsalva and Toynbee manoeuvres	Pre-operative	30	20
		8 weeks post-operative	14	36
Akyildiz (2017)^13^	Valsalva and Toynbee manoeuvres	Pre-operative	16	34
		30 days post-operative	10	40
		90 days post-operative	4	46

Kaya *et al*.^12^ also reported similar findings utilising the Valsalva and Toynbee manoeuvres, with a pre-operative ‘good’ Eustachian tube function outcome of 20 participants (40 per cent), which increased to 36 participants (72 per cent) with a ‘good’ Eustachian tube function outcome at the 8-week follow up post-surgery. Kaya *et al*.^12^ also used the same criteria as Salvinelli^4^ to report ‘poor’ and ‘good’ Eustachian tube function outcomes. Likewise, Akyildiz *et al*.^13^ also used the same criteria and manoeuvres as Salvinelli^4^ and Kaya *et al*.^12^ to report Eustachian tube functions with a pressure difference of 10 daPa or more reported as ‘normal’ and a pressure difference of less than 10 daPa reported as ‘dysfunctional’ following intervention.

Akyildiz *et al*.^13^ reported that 17 participants (68 per cent) had a ‘normal’ Eustachian tube function pre-operatively, which increased to 20 participants (80 per cent) 1 month post-operatively, and further increased to 23 participants (92 per cent) 3 months post-operatively.

### Eustachian Tube Dysfunction Questionnaire-7

Eustachian tube dysfunction symptoms are assessed using the Eustachian Tube Dysfunction Questionnaire-7, which ranges from a score of 7 to a maximum score of 49. A higher score on the questionnaire indicates more severe dysfunction. Lima e*t al*.,^3^ Lee *et al*.^2^ and Daum *et al*.^14^ used the questionnaire to evaluate Eustachian tube function following nasal surgery. Lima *et al*.^3^ reported a pre-operative median score of 14, which decreased after the 3- to 6-month follow up to 11 post-operatively (*p* < 0.001). Lee *et al*.^2^ revealed a pre-operative mean score of 12.76 ± 6.62, which dropped to 8.47 ± 2.66 3 months after surgery (*p* < 0.001).

Daum *et al*.^14^ observed a decrease in mean scores from 23.3 ± 7.6 pre-operatively in a total of 97 patients analysed to 19.1 ± 9.1 at 1 week post-operation (*n* = 82, *p* = 0.002), 16.5 ± 8.0 at 1 month post-operation (*n* = 68, *p* < 0.001), 16.2 ± 7.8 at 3 months post-operation (*n* = 39, *p* < 0.001) and 16.7 ± 10.4 at 6 months post-operation (*n* = 24, *p* < 0.001).

### Tympanometry

Alshareef *et al*.^8^ used tympanograms to measure Eustachian tube dysfunction and reported separate results for each ear. In the right ear, the pre-operative distribution of tympanograms included type A in 12 cases (66.7 per cent), type B in 2 cases (11.1 per cent) and type C in 4 cases (22.2 per cent). After 30 days post-operation, type A tympanograms increased to 16 cases (88.8 per cent), type B remained stable in 1 case (11.1 per cent) and type C decreased in 1 case (11.1 per cent). Similarly, the left ear exhibited pre-operative tympanograms with type A in 12 cases (66.7 per cent), type B in 2 cases (11.1 per cent) and type C in 4 cases (22.2 per cent). At 30 days post-operation, type A tympanograms remained stable in12 cases (66.7 per cent), with type B in 2 cases (11.1 per cent) and Type C in 4 cases (22.2 per cent). At the 6-month follow up, type A tympanograms increased in 17 cases (94.4 per cent) in the left ear and type C decreased in 1 case (5.6 per cent).

### Nasal Obstruction Symptom Evaluation score

The Nasal Obstruction Symptom Evaluation score quantifies the severity of nasal obstruction symptoms, with higher scores indicating greater obstruction. Kaya *et al*.^12^ reported a pre-operative mean Nasal Obstruction Symptom Evaluation score of 12.48 ± 4.78, which significantly decreased to 7.56 ± 3.4 at 8 weeks post-operatively (*p* < 0.001). Lima *et al*.^3^ also reported Nasal Obstruction Symptom Evaluation scores with a pre-operative median (interquartile range) score of 60 (30) and a post-operative score of 20 (5).

### Meta-analysis

Meta-analysis was performed, focusing primarily on two outcomes: Eustachian Tube Dysfunction Questionnaire-7 scores and Eustachian tube function tests.

**Figure 3.**
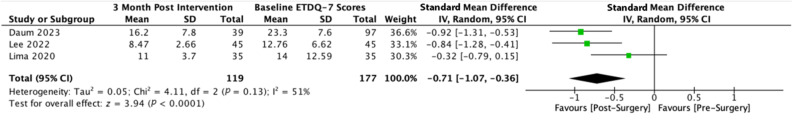
ETDQ-7 Scores (With Lima, 2020).

**Figure 4.**
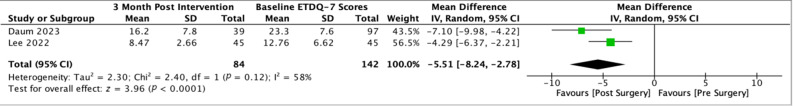
ETDQ-7 Scores (w/o Lima, 2020).

A total of three studies^2^^,^^3^^,^^14^ reported data on Eustachian Tube Dysfunction Questionnaire-7 scores, two^2^^,^^14^ of which reported data as means and standard deviations, while the third^3^ reported data as median and interquartile range. We used the methods described in Deeks *et al*.^15^ and Wan *et al*.^16^ to estimate the mean and standard deviation from the median and interquartile range. Data from these 3 studies returned a pooled standard mean difference of −0.71 (95 per cent confidence interval (CI) = −1.07 to −0.36), revealing a statistically significant decrease in Eustachian Tube Dysfunction Questionnaire-7 scores post-surgery (Figure 3). However, remaining cognisant of the limitations of transforming non-normal data to normal, we also ran a separate analysis for the two studies reporting data in means and standard deviations. This pooled analysis also revealed a statistically significant decrease in Eustachian Tube Dysfunction Questionnaire-7 scores post-surgery with a mean difference of −5.51 (95 per cent CI = −8.24 to −2.78) (Figure 4).

**Figure 5.**
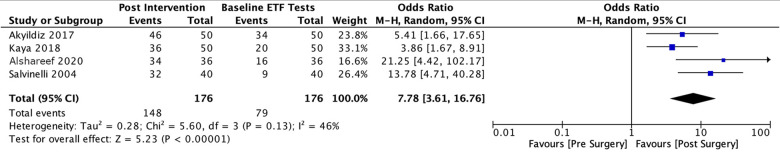
ETF Tests (Maximum Follow-Up in Each Study).

A total of four included studies^4^^,^^8^^,^^11^^,^^12^ reported on Eustachian tube function tests, with all four describing Eustachian tube function as either ‘good’ or ‘poor’, or ‘normal’ or ‘dysfunctional’, based on the change in tympanometric peak pressure following the intervention described above. Three of these four studies reported the exact criteria that they used to develop these categories,^4^^,^^12^^,^^13^ which were the same in all three studies, while the fourth study^8^ did not report the specific criterion used to determine ‘good’ or ‘poor’ Eustachian tube function. All four studies reported data at slightly different follow-up intervals, therefore we conducted multiple meta-analyses to capture the pooled data. The first analysis was run at the maximum follow-up interval conducted in each study with the three-month follow-up interval by Akyildiz *et al*.,^13^ the eight-week follow-up interval by Kaya *et al*.,^12^ the six-month follow-up interval by Alshareef *et al*.^8^ and the three-month follow-up interval by Salvinelli.^4^ The pooled data revealed a statistically significant odds ratio of 7.78 (95 per cent CI = 3.61 to 16.76), indicating a significant increase in ‘good’ outcome Eustachian tube function tests post-intervention (Figure 5).

**Figure 6.**
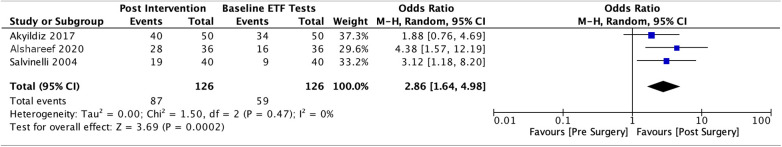
ETF Test (Followed-Up at 1 Month).

**Figure 7.**
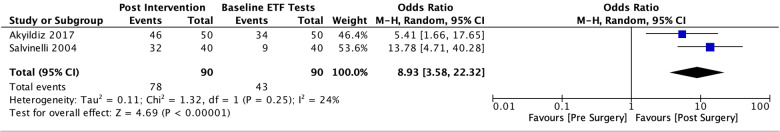
ETF Tests (Followed-Up at 3 Months).

**Figure 8.**
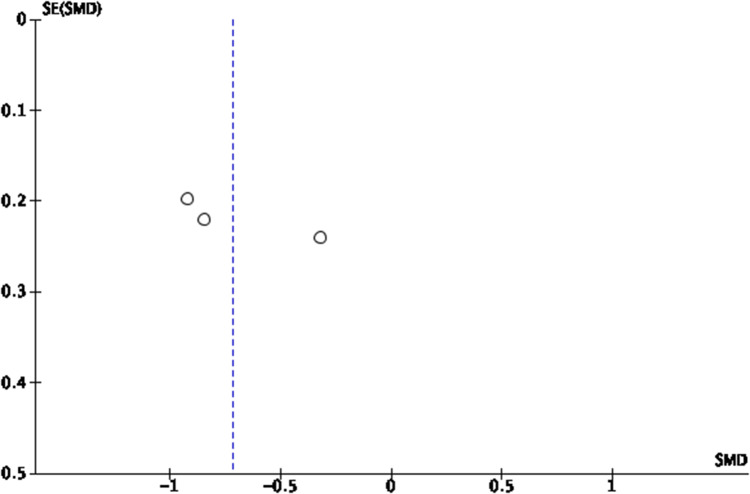
Funnel plot of comparison, outcome - ETDQ-7 scores (with Lima, 2020).

**Figure 9.**
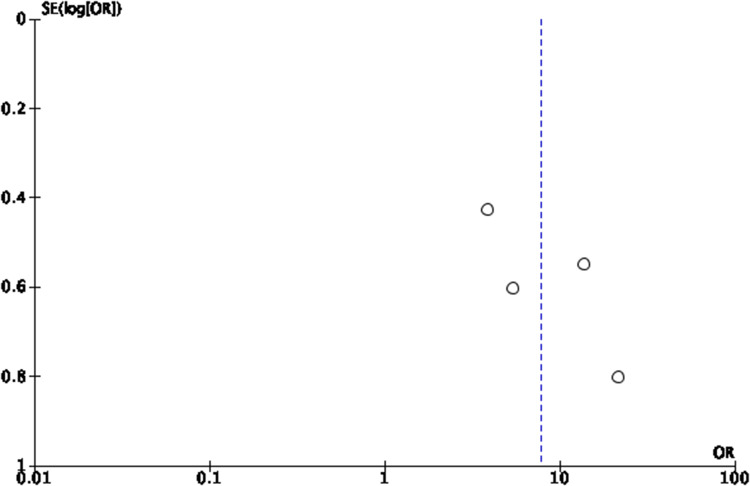
Funnel plot of comparison, outcome - ETF Tests at Maximum Follow-Up Interval in Each Study.

Another analysis was conducted for Eustachian tube function tests reported at a follow-up interval of 1 month with 3 studies^4^^,^^8^^,^^13^ pooled, which revealed a statistically significant odds ratio of 2.86 (95 per cent CI = 1.64 to 4.98) (Figure 5). This was echoed by our third analysis conducted for Eustachian tube function tests reported at a follow-up interval of 3 months with 2 studies^4^^,^^13^ pooled, which also revealed a statistically significant odds ratio of 8.93 (95 per cent CI = 3.58 to 22.32). (Figure 7) Funnel plots for Eustachian Tube Dysfunction Questionnaire-7 using standardised mean differences and Eustachian tube function tests using odds ratios are shown in Figures 8 and 9.

## Discussion

Chronic rhinosinusitis and a deviated nasal septum have been highlighted in previous studies to frequently coexist with Eustachian tube dysfunction, leading to symptoms such as ear pressure, aural fullness, tinnitus and hearing loss.^7^^,^^17^ Surgical interventions targeting the nasal cavity, such as FESS and septoplasty, have been proposed as potential treatments for alleviating Eustachian tube dysfunction symptoms by addressing underlying nasal pathologies.^17^^–^^19^ In this systematic review and meta-analysis, we demonstrated the positive impact of nasal surgery on various outcomes related to Eustachian tube dysfunction, including improvements in Eustachian Tube Dysfunction Questionnaire-7 scores, tympanogram findings, Nasal Obstruction Symptom Evaluation scores and Eustachian tube function tests. This aligns with the findings of authors in existing literature through limited case series or comparative studies.

Nasal surgery, particularly FESS and septoplasty, has shown promising results in improving Eustachian tube dysfunction symptoms as assessed by the Eustachian Tube Dysfunction Questionnaire-7. The Eustachian Tube Dysfunction Questionnaire-7 is a validated tool used to quantify the severity of Eustachian tube dysfunction symptoms, including ear blockage, popping and pain.^20^^–^^22^ Studies have reported significant reductions in Eustachian Tube Dysfunction Questionnaire-7 scores following nasal surgery, indicating an improvement in subjective Eustachian tube dysfunction symptoms.^2^^,^^3^^,^^14^ These findings suggest that addressing nasal pathologies through surgical interventions can lead to symptomatic relief in patients with Eustachian tube dysfunction. Arguably, this is the most relevant measurement of a successful intervention because it directly reflects the improvement in patients’ symptoms.

The objective assessment of Eustachian tube function, such as sonotubometry or tubomanometry, also offers valuable insights into Eustachian tube dysfunction pathophysiology and treatment response. Nasal surgery has been associated with improvements in Eustachian tube function tests, including increased Eustachian tube opening pressure and duration.^4^^,^^23^^,^^24^ These objective findings corroborate subjective symptom improvements and support the role of nasal surgery in restoring Eustachian tube function.

Tympanometry is a valuable tool for assessing middle-ear function and can provide insights into Eustachian tube dysfunction-related changes in middle-ear pressure and compliance. Studies evaluating tympanometric findings before and after nasal surgery have reported improvements in tympanogram patterns, including resolution of negative pressure and type C tympanograms associated with Eustachian tube dysfunction.^4^^,^^25^ These improvements suggest that nasal surgery may normalise middle-ear function by restoring Eustachian tube patency and equalising middle-ear pressure.

Looking ahead, future research should focus on elucidating the mechanisms underlying the observed improvements in Eustachian tube dysfunction symptoms and objective measures following nasal surgery. Studies investigating the effects of nasal surgery on Eustachian tube anatomy and physiology using imaging modalities such as computed tomography and magnetic resonance imaging can provide valuable insights into the structural changes associated with Eustachian tube dysfunction resolution.^26^^–^^28^ Additionally, longitudinal studies assessing long-term outcomes and predictors of treatment response can help refine patient selection criteria and optimise treatment algorithms for Eustachian tube dysfunction management.^29^^,^^30^

Incorporating treatment of nasal symptoms into the management of Eustachian tube dysfunction represents a holistic approach to addressing the multifactorial nature of this condition. By targeting nasal inflammation and mucosal oedema, interventions such as intranasal corticosteroids and saline irrigation can complement Eustachian tube dysfunction-specific treatments and enhance overall treatment efficacy.^31^^,^^32^ Furthermore, adjunctive therapies such as balloon dilation of the Eustachian tube may synergise with nasal surgery to optimise Eustachian tube dysfunction symptom relief.^33^^,^^34^

- Nasal surgery holds promise as a therapeutic option for patients with Eustachian tube dysfunction associated with a deviated nasal septum, offering significant symptom relief and improved quality of life
- Incorporating treatment of nasal symptoms into the management of Eustachian tube dysfunction represents a holistic approach to addressing the multifactorial nature of Eustachian tube dysfunction
- By integrating the treatment of nasal symptoms into the management of Eustachian tube dysfunction, clinicians can adopt a comprehensive approach to addressing the underlying pathologies contributing to this condition
- Looking ahead, future research should focus on elucidating the mechanisms underlying the observed improvements in Eustachian tube dysfunction symptoms and objective measures following nasal surgery
- The treatment algorithms should also be refined and novel therapeutic interventions explored to further enhance Eustachian tube dysfunction management strategies

## Conclusion

Nasal surgery has demonstrated promising results as a therapeutic option for patients with Eustachian tube dysfunction and a deviated nasal septum, offering significant symptom relief and improved quality of life. Through the integration of the treatment of nasal symptoms into the management of Eustachian tube dysfunction, clinicians can adopt a comprehensive approach to addressing the underlying pathologies contributing to this condition. Future research endeavours should focus on elucidating the mechanisms of action of nasal surgery, refining treatment algorithms and exploring novel therapeutic interventions to further enhance Eustachian tube dysfunction management strategies.
